# Relationship between markers of inflammation and hemodynamic stress and death in patients with out-of-hospital cardiac arrest

**DOI:** 10.1038/s41598-021-88474-3

**Published:** 2021-05-11

**Authors:** Thomas A. Zelniker, Ziya Kaya, Eva Gamerdinger, Sebastian Spaich, Jan Stiepak, Evangelos Giannitsis, Hugo A. Katus, Michael R. Preusch

**Affiliations:** 1 Division of Cardiology, Medical University of Vienna, Waehringer Guertel 18-20, Vienna, Austria; 2 Department of Cardiology, Angiology, and Pneumology, University Hospital Heidelberg, Im Neuenheimer Feld 410, 69120 Heidelberg, Germany; 3 Department of Cardiology, Robert-Bosch-Hospital, Stuttgart, Germany; 4 German Center for Cardiovascular Research (DZHK), Partner Site Heidelberg/Mannheim, Heidelberg, Germany

**Keywords:** Biomarkers, Cardiology, Health care, Medical research, Risk factors

## Abstract

Biomarkers that reflect hemodynamic stress, inflammation, extracellular matrix remodeling, angiogenesis, and endothelial dysfunction may improve risk stratification and add valuable pathobiological insight in patients with out-of-hospital cardiac arrest (OHCA). In total, 120 patients with OHCA who survived at least 48 h after return of spontaneous circulation were consecutively included in the present analysis. Concentrations of 30 biomarkers were measured simultaneously using a multi-panel biomarker assay. Cox regression models were adjusted for age, sex, estimated glomerular filtration rate, lactate concentration, bystander resuscitation, initial cardiac rhythm, and type of targeted temperature management. Overall, 57 patients (47.5%) had a favorable neurological outcome (Cerebral Performance Category ≤ 2) at 30 days, while palliative care was initiated in 49 patients (40.8%), and 52 patients (43.3%) died. After correction for multiple testing with Bonferroni-Holm, 8 biomarkers (including A*ngiopoietin-2*, P*rocalcitonin*, R*esistin*,* IL-4Rα*,* MMP-8*,* TNFα*,* Renin,* and *IL-1α*) were significantly associated with all-cause death. After multivariable adjustment, only angiopoietin-2 (Adjusted (Adj) hazard ratio (HR) per 1-unit increase in standardized biomarker concentrations 1.52 (95% CI 1.16–1.99)) and renin (Adj HR 1.32 (95% CI 1.06–1.65) remained independently associated with an increased risk of death. The discriminatory performance indicated good performance for angiopoietin-2 (area under the curve (AUC): 0.75 (95% CI 0.66–0.75) and was significantly higher (*P* = 0.011) as compared with renin (AUC: 0.60, 95% CI 0.50–0.60). In conclusion, angiopoietin-2 was significantly associated with all-cause mortality in patients with OHCA who survived the first 48 h and may prove to be useful for risk stratification of these patients.

## Introduction

Despite significant advances in post-cardiac arrest management over the last decades, including implementation of targeted temperature management^1^ (TTM) and the proposal of specialized centers for post-cardiac arrest patients^2^, outcomes of patients with out-of-hospital cardiac arrest (OHCA) after return-of-spontaneous circulation (ROSC) remain poor^3^. Regardless of the underlying etiology of cardiac arrest (CA), these patients are at high risk of developing severe complications leading to multi-organ failure and death due to myocardial dysfunction, systemic inflammatory and ischemic/reperfusion response, and anoxic brain injury^4^. In addition to determining the cause of CA and implementing disease-specific interventions, mitigation of these risks remains imperative. This complex syndrome, which has been termed post-cardiac arrest syndrome, is characterized by several different pathophysiological processes, including hemodynamic stress, endothelial dysfunction, and inflammation. Although some markers of hemodynamic stress have been found to be associated with an increased risk of death in patients with cardiac arrest^5,6^, surrogate markers of inflammation and of endothelial function and structure have been less well studied. With this variety of pathobiological pathways involved, we hypothesized that a panel of biomarkers that reflect hemodynamic stress, inflammation, extracellular matrix remodeling, angiogenesis, and endothelial dysfunction might improve risk stratification and add valuable pathobiological insight. Mortality due to hemodynamic compromise and withdrawal of care dominates mechanisms of early post-cardiac arrest mortality, while other underlying pathophysiologic processes, including septic shock, are more likely to occur later. Consequently, we aimed to study a multi-biomarker panel reflecting different pathways in patients with OHCA and ROSC who survived at least 48 h.

## Materials and methods

### Study population

The present exploratory analysis is a biomarker study from the Heidelberg Resuscitation Registry^5,6^. The Heidelberg Resuscitation Registry is an ongoing prospective registry in a tertiary hospital setting that consecutively enrolls patients with OHCA after successful ROSC^5,6^. Eligible patients for this study were required to have survived at least 48 h after ROSC and to have available blood samples on admission and at 48 h. Patients transferred from other hospitals were not included in this study. The protocol was approved by the institutional review board (Ethics board of the University Heidelberg, S388-2011). All research was performed following relevant guidelines/regulations and written informed consent was obtained from all patients or their legal representatives. As described previously^5,6^, all patients were treated according to standard operating procedures based on current guidelines and literature. Targeted temperature management was performed using an endovascular cooling device (Coolgard 3000/ICY catheter, Zoll Medical Corp., USA), and a target temperature of 33 °C was maintained for 24 h. Patients not treated with TTM using an endovascular cooling device were cooled externally using basic tools such as ice packs and cold saline solution targeted to maintain a temperature at 36 °C. An endovascular cooling device was not implemented in case of unavailability of a device at time of initial presentation or contraindications (e.g. severe bleeding, or regained consciousness prior to presentation)^5,6^.

### Laboratory assessment and biomarker testing

Blood samples were collected on admission and 48 h after admission in serum-separating and EDTA-anticoagulated plastic tubes; serum and plasma were isolated within 60 min of sample acquisition and then stored at -80 °C until measurement. A panel of 30 different biomarkers (angiopoietin-1, angiopoietin-2, endothelin-1, eotaxin, granzyme B, interferon (IFN) γ, interleukin (IL)-1α, IL-1β, IL-4Rα, IL-6, IL-8, IL-9, IL-10, IL-12p70, IL-17A, IL-21, IL-23, interferon gamma-induce protein (IP)-10, monocyte chemoattractant protein (MCP)-1, macrophage inflammatory protein (MIP)-1α, MIP-1β, matrix metallopeptidase (MMP)-8, mucin (MUC)-16, placenta growth factor (PIGF), procalcitonin, renin, resistin, tumor necrosis factor (TNF)α, TNF-like weak inducer of apoptosis (TWEAK), and vascular endothelial growth factor (VEGF)) was measured simultaneously in serum samples on a research use only, magnetic bead-based multiplex assay for the Luminex platforms^7^.

In addition to the above-described study cohort, this multimarker panel was measured in 24 clinically healthy control subjects (18 men and 6 women) as a reference population. As described previously^8^, the inclusion criteria were clinically healthy men and women with a body mass index between 20 and 35 kg/m^2^, who had to have been stable in the previous 4 weeks (a variance of ± 2 kg was accepted). Clinically healthy individuals had to have an unremarkable physical examination and normal structural and functional cardiac parameters defined as a left ventricular ejection fraction ⩾55%, normal right ventricular function, normal valve function, normal dimensions and volumes of all heart chambers, and a normal stress test using either dynamic echocardiography or adenosine or dobutamine stress MRI. All biomarker testing was performed by personnel blinded to clinical outcomes in the affiliated research laboratories of the University Hospital Heidelberg. High-sensitivity C-reactive protein (hsCRP) was measured as part of the department-specific standard operating procedure in OHCA patients in the clinical core lab of the University Hospital of Heidelberg on admission but not at 48 h.

### Endpoints

The primary endpoint for this biomarker analysis was time to all-cause mortality. Initiation of palliative therapy was collected from the discharge letter, but specific causes of death were otherwise not adjudicated. All patients were followed for at least 30 days (Supplemental Fig. 1). Moreover, we assessed the cerebral performance category (CPC) score at 30 days.

### Statistical analysis

We report the median observation time and the median follow-up using the reverse Kaplan–Meier estimator^9^. Biomarkers were analyzed as continuous variables as well as categorized using medians, quartiles (Q) and dichotomized using Q4 versus Q1 to Q3. Biomarker levels were compared between surviving and deceased patients with OHCA and an apparently healthy patient cohort using Bonferroni-Holm corrected Wilcoxon Rank sum tests when the Kruskal Wallis omnibus test was significant. The association between biomarker levels measured at 48 h (included as a standardized variable) and all-cause death was first analyzed using unadjusted Cox proportional hazards regression models. Biomarkers that met statistical significance after correction for multiple testing using the Bonferroni-Holm method, which controls the family-wise error, were further evaluated in adjusted Cox models that included the following variables: age, sex, estimated glomerular filtration rate (at 48 h), lactate concentration (at 48 h), bystander resuscitation, presence of shockable rhythm as the first monitored heart rhythm, and type of targeted temperature management (33**°** versus 36°Celsius). Subgroup analyses were conducted stratified by presence of bystander resuscitation (yes vs. no), type of first monitored ECG rhythm (shockable versus non-shockable) presumed etiology of cardiac arrest (cardiac versus non-cardiac etiology), and type of targeted temperature management (33 °C versus 36 °C). Tests for heterogeneity were determined by including an interaction term in the unadjusted models. The relationship between biomarkers and the CPC score were assessed using ordinal logistic regression models. Kaplan Meier estimates are reported at 30 days and compared using the logrank test. The discriminatory performance was assessed by the c-statistic^10^. To explore the relationship between biomarker concentrations and all-cause mortality, we fitted adjusted Cox models with biomarker concentrations entered as natural splines. The degrees of freedom were chosen according to the Akaike Information Criterion (AIC) using the R package ‘smoothHR’^11^. All analyses were performed using R (Version 4.0.3)^12^. Two-sided *P* values < 0.05 were considered to indicate statistical significance.

## Results

### Study population

Among 383 patients who were admitted between 26 May 2013 and 27 June 2017, 120 patients were identified to meet the inclusion criteria and included in the present study (Supplemental Fig. 1). The median observation time was 26 days (interquartile range (IQR) 10 – 373 days)and the median follow-up time using the reverse KM estimator was 354 days (IQR 97–684 days). The patients’ median age was 64.5 years, and 29 patients (24.2%) were female. Eighty-three patients (69.2%) received bystander-initiated CPR (Table 1). The majority of patients had shockable rhythm as the first monitored heart rhythm (n = 73, 59.8%) and a presumed cardiac etiology (n = 90, 75%) as the cause of cardiac arrest.

**Table 1:** Patient characteristics.

Demographic characteristics	(N = 120)
Age, median [IQR]	64.5 [51.0, 73.0]
Female sex, n (%)	29 (24.2%)
First monitored heart rhythm (shockable), n (%)	73 (59.8%)
Bystander-initiated cardiopulmonary resuscitation, n (%)	83 (69.2%)
Time to ROSC (min), median [IQR]	21.0 [15.0, 30.0]
Cardiac etiology (yes), n (%)	90 (75%)
SAPS II score on admission, median [IQR]	64.0 (57.5, 71.0)
Targeted temperature management at 33 °C, n (%)	97 (80.8%)
eGFR at 48 h (ml/min/1.73 m^2^), median [IQR]	56.9 [30.9, 92.2]
Cerebral performance category ≤ 2 at 30 days, n (%)	57 (47.5%)
Palliative therapy, n (%)	49 (40.8%)
Death	52 (43.3%)

Continuous variables are reported as median and interquartile range.

*eGFR* estimated glomerular filtrations rate, *ROSC* return of spontaneous circulation, *SAPS II* Simplified Acute Physiology Score II, *CPR* cardiopulmonary resuscitation.

Overall, 57 patients (47.5%) had a favorable neurological outcome (CPC ≤ 2) at 30 days, while palliative care was initiated in 49 patients (40.8%), and 52 patients (43.3%) died. Patients who died were more likely to be older, male, and have a higher SAPS II score on admission but had a lower eGFR (Supplemental Table 1).

### Biomarker levels at 48 h post OHCA

The median biomarker levels measured at 48 h in patients with OHCA and the correlation matrix are shown in Supplemental Tables 2 and 3. Levels of all biomarkers, except those of IL-9 and PlGF, differed significantly between deceased and survived patients with OHCA, and an apparently healthy patient population (Supplemental Fig. 2, Supplemental Table 2).

### Association between inflammatory biomarkers and all-cause death in patients with OHCA

After correction for multiple testing with Bonferroni-Holm, eight biomarkers were significantly associated with all-cause death in unadjusted Cox regression models (Unadjusted hazard ratios (HR) per 1-unit increase in standardized biomarker concentrations: A*ngiopoietin-2:* HR 1.79 (95% CI 1.46–2.19), *IL-4Rα*: HR 1.52 (95% CI 1.27–1.83), *MMP-8*: HR 1.36 (95% CI 1.15–1.61), P*rocalcitonin*: HR 1.73 (95% CI 1.38–2.18), *Renin:* HR 1.36 (95% CI 1.12–1.64), and *IL-1α:* HR 1.29 (95% CI 1.10–1.52), R*esistin*: HR 2.09 (95% CI 1.54–2.84), *TNFα:* HR 1.29 (95% CI 1.11–1.49); Supplemental Table 3). Similar findings were observed for biomarker levels on admission (Supplemental Tables 5 and 6), but neither biomarker concentrations on admission nor change in biomarker levels were significantly associated with death when added to biomarkers measured at 48 h.

**Figure 1:**
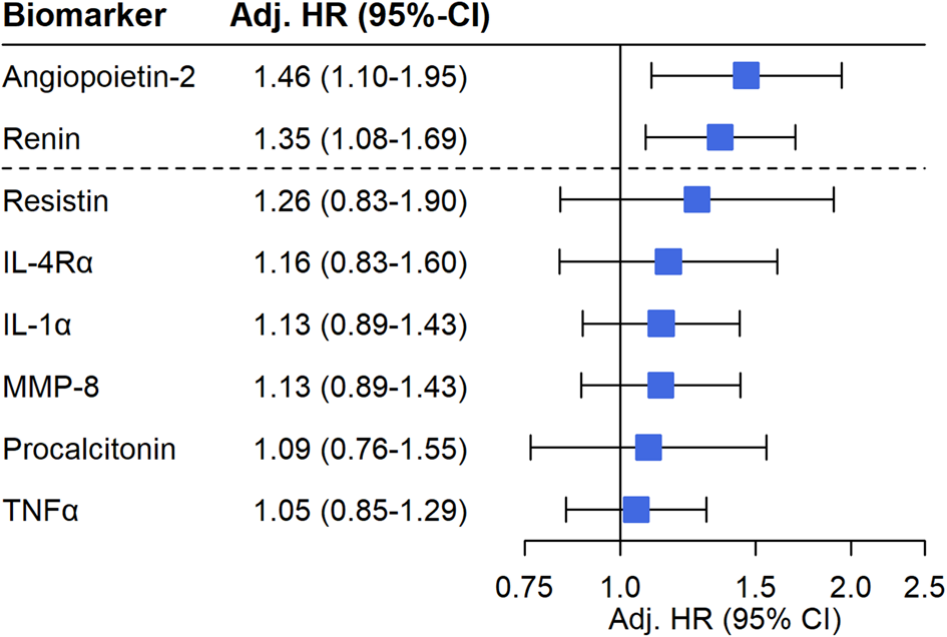
Adjusted hazard ratios for biomarkers per 1-unit increase in standardized biomarker levels and all-cause death. The models were adjusted for age, sex, estimated glomerular filtration rate at 48 h, lactate levels at 48 h, bystander resuscitation, presence of shockable rhythm as the first monitored heart rhythm, and type of targeted temperature management (33° versus 36°Celsius).

**Figure 2:**
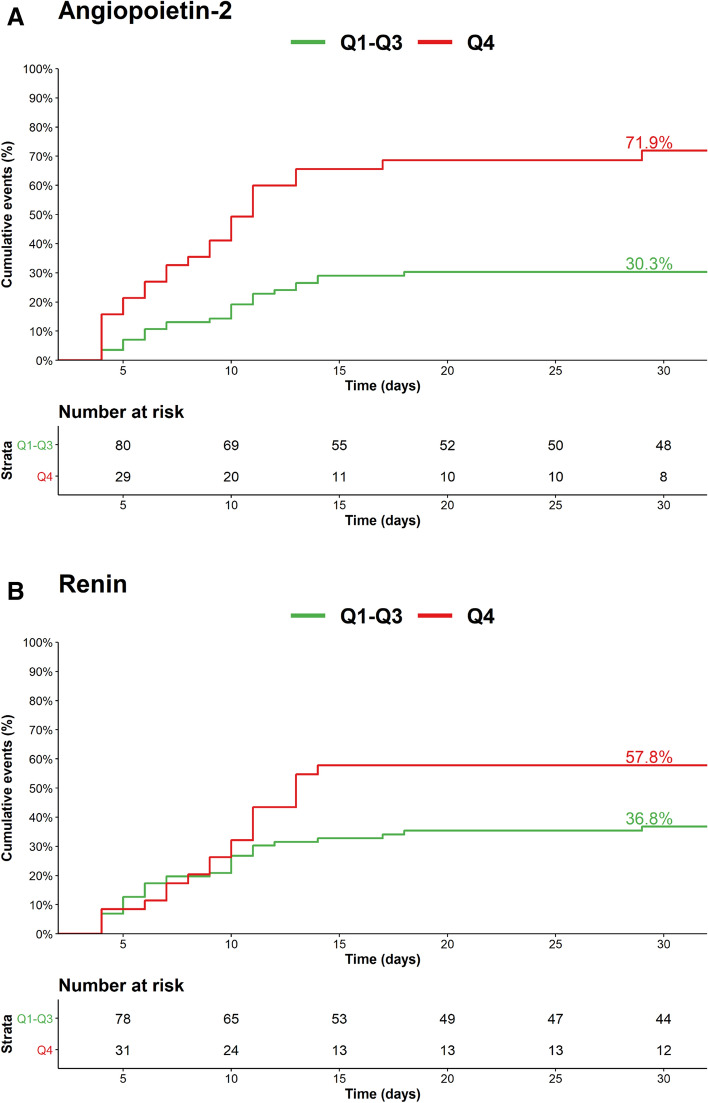
Cumulative event rates through 30 days for all-cause death stratified by the top quartile (Q4) versus Q1-Q3 of angiopoietin-2 (**A**) and renin (**B**). (**A**) Angiopoietin-2: Q1-Q3 ≤ 13.4 pg/ml versus Q4 > 13.4 pg/ml, Logrank *P* < 0.001. (**B**) Renin: Q1-Q3 ≤ 1.96 pg/ml versus Q4 > 1.96 pg/ml, Logrank *P* = 0.060.

**Figure 3:**
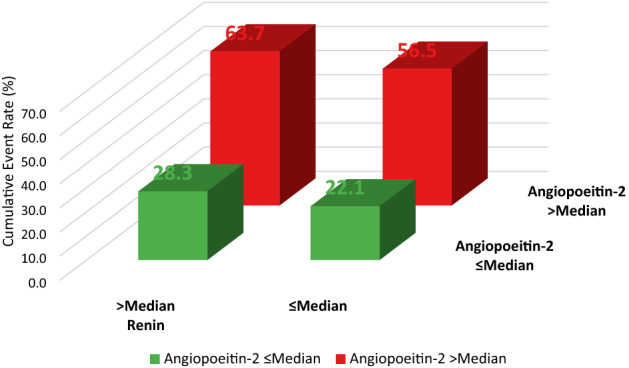
Kaplan–Meier event rates at 30 days stratified by the median of angiopoietin-2 and renin for all-cause death. Renin > median: Angiopoietin-2 ≤ median versus  > median, Logrank *P* = 0.001, Renin ≤ median: Angiopoietin-2 ≤ median versus  > median; Logrank *P* = 0.009. Angiopoietin-2 > median: Renin ≤ median versus  > median, Logrank P = 0.10, Angiopoietin-2 ≤ median: Renin ≤ median versus  > median; Logrank *P* = 0.80.

**Figure 4:**
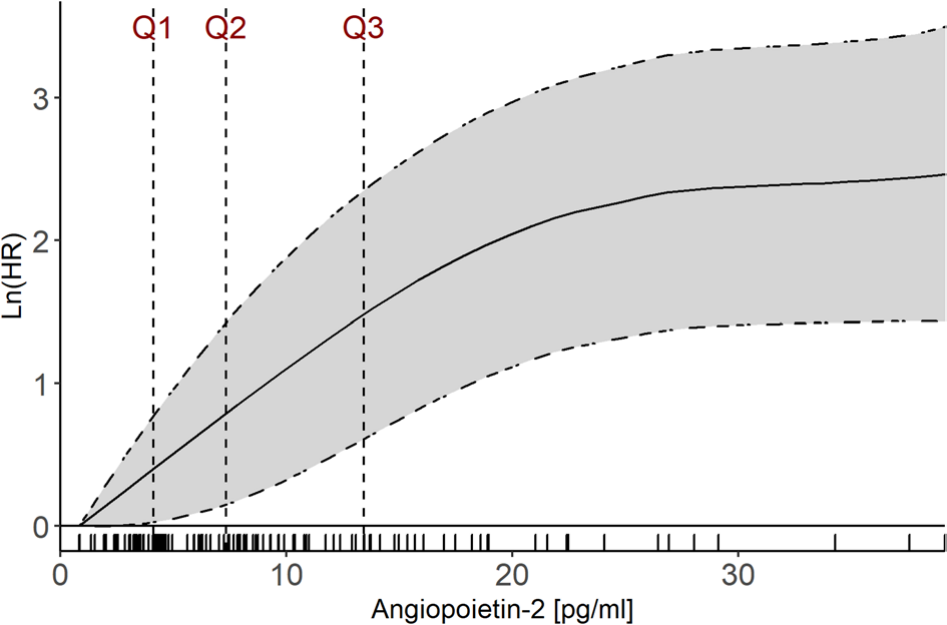
Estimated adjusted log-hazard ratios for all-cause mortality events in relation to continuous serum angiopoietin-2 levels modeled with penalized regression splines. Note the dashed vertical lines indicating the 25th, 50th and 75th percentiles. The rug plot illustrates the marginal distributions of angiopoietin-2 concentration.

After multivariable adjustment, only *angiopoietin-2* (Adjusted (Adj) HR per 1-unit increase in standardized biomarker concentrations 1.46 (95% CI 1.10–1.95)) and *renin* (Adj HR per 1-unit increase in standardized biomarker concentrations 1.35 (95% CI 1.08–1.69) remained independently associated with an increased risk of all-cause mortality (Fig. 1). This relationship remained virtually identical after further adjustment for patients’ baseline *Simplified Acute Physiology Score II (SAPS II)* score (Supplemental Fig. 3) and baseline high-sensitivity troponin T and baseline high-sensitivity CRP (Supplemental Fig. 4). The Kaplan Meier curves separated early (< 5 days) for angiopoetin-2 (Fig. 2A) while for renin (Fig. 2B) the division occurred delayed after nearly 2 weeks. The event rates stratified by the median of both angiopoietin-2 and renin indicated angiopoietin-2 to be of complementary nature for renin (both Logrank *P* values < 0.01) but not vice versa (both Logrank *P* values > 0.10) (Fig. 3). In a bivariable model of angiopoietin-2 and renin, as well as in the multivariable adjusted model including the full set of covariates, only angiopoietin-2 remained statistically significant. Angiopoietin-2 modeled with adjusted natural smoothed regression splines, indicated a consistent nearly linear increase in the hazard for all-cause death from 1.5 to 40 pg/ml of biomarker concentrations (Fig. 4). Moreover, both biomarkers angiopoietin-2 and renin were significantly associated with worse CPC score (angiopoietin-2: odds ratio (OR) 2.51, 95% CI 1.25–6.13; renin: OR 2.22, 95% CI 1.28–4.67).

### Discriminatory performance

The discriminatory performance indicated good performance for angiopoietin-2 (area under the curve (AUC): 0.75 (95% CI 0.66–0.75) and was significantly higher (Δ 0.15, *P* = 0.011) compared with the AUC for renin (AUC: 0.60, 95% CI 0.50–0.60; Table 2). The addition of the two biomarkers to the clinical covariates used for adjustment and the baseline SAPS II score resulted in a numerical but non-significant improvement of the AUC for the discrimination of death (AUC 0.94 versus 0.91, Δ = 0.03, *P* value 0.12).

**Table 2:** C-statistics with the respective area under curve (AUC) and 95% confidence intervals (95% CI) for all-cause mortality.

Biomarker	AUC	95% CI
Angiopoietin-2	0.75	0.66–0.75
Procalcitonin	0.75	0.66–0.75
Resistin	0.74	0.65–0.74
IL-4Rα	0.71	0.62–0.71
IL-1α	0.68	0.58–0.68
MMP-8	0.67	0.58–0.67
TNFα	0.65	0.55–0.65
Renin	0.60	0.50–0.60

### Subgroup analyses

The association between angiopoietin-2 and renin, respectively, and the risk of death was consistent (*P*-interaction > 0.10) across different tested subgroups, including the presence of bystander resuscitation, presumed etiology of cardiac arrest, type of first monitored ECG rhythm, and type of targeted temperature management. However, there was a significant interaction (*P* value for interaction 0.046) for renin based on the type of first monitored ECG rhythm. The association tended to be stronger in patients with shockable rhythm (HR 1.73, 95% CI 1.29–2.33) as compared with patients with non-shockable ECG (HR 1.15, 95% CI 0.87–1.52; *P* value for interaction 0.046). Otherwise, there were no subgroup differences for renin.

## Discussion

In this study examining a panel of multiple novel and clinically established biomarkers, angiopoietin-2 emerged as a biomarker that was strongly associated with all-cause mortality in patients with OHCA.

Early risk stratification and neurological prognostication of patients with OHCA remains challenging. Clinical examination and electrophysiological measurements are impeded by sedation during targeted temperature management. Therefore, biomarkers can be helpful tools that provide diagnostic and/or prognostic information and give insight into pathobiological mechanisms in this early phase of treatment. In patients with OHCA, multiple pathophysiological processes are involved in the postcardiac arrest phase. Critical hemodynamic stress often manifests and offers a profound challenge predominantly in the early post-cardiac arrest period, while inflammatory processes may occur with some delay. The restoration of blood flow to all organs is followed by a global ischemia–reperfusion insult that promotes systemic inflammatory response syndrome. Shock further enhances proinflammatory cytokines that lead to inappropriate vasodilation, impaired vascular barrier function, and capillary leakage with direct cellular damage and edema. However, hemodynamic impairment and inflammation may alternate or coincide.

The tested biomarker panel reflects several critical pathophysiological processes present in patients after ROSC, including hemodynamic stress, inflammation, and vascular integrity. In the current analysis, angiopoietin-2 emerged as a possibly valuable risk marker. Angiopoietin-2 had the highest discriminatory performance among the tested biomarkers. Although the effects of angiopoietin-2 are context-dependent and may exert even opposite effects depending on the hormonal milieu, the angiopoietin-Tie2 ligand-receptor system has been shown to play an essential role in the endothelial barrier function^13,14^. As a marker of vascular integrity, angiopoietin-2 may therefore be associated with the presence of edema reflecting conditions with unfavorable prognosis such as capillary leak syndrome or edema of the brain in post-cardiac arrest patients. In addition to its pro-angiogenetic properties, angiopoietin-2 has also been implicated in inflammatory processes^15^. Previous studies have shown higher angiopoietin-2 concentrations in patients with decompensated heart failure and cardiogenic shock^16–18^. Furthermore, angiopoietin-2 levels have been found to be increased in patients with sepsis^19^ and a mendelian randomization analysis suggested a possible causal role of plasma angiopoietin-2 in the development of acute respiratory distress syndrome^20^. These data, therefore, add to the growing body of evidence that angiopoietin-2 may have pathophysiological relevance and a prognostic role in critically ill patients.

### Limitations

Several limitations should be addressed: The sample size is limited, and patients were only recruited at one center. Moreover, generalizability is limited as only OHCA patients who survived the first 48 h were included in this study. The addition of angiopoietin-2 and renin did not result in a significant improvement of risk discrimination compared with a comprehensive clinical model, though it is important to note that the comparisons of very well-performing models are low-power procedures. Furthermore, we did not assess the specific cause of death. Also, other important established biomarkers, including hsCRP and high-sensitivity troponin T were only measured on admission but not at 48 h.

Although these findings require external validation, the present data add to the growing body of evidence that employment of biomarkers can be helpful to improve risk stratification in patients with OHCA. Future research should focus on the clinical utility of multimarker approaches that reflect different pathophysiological pathways, the development and validation of easy implementable risk scores, and address whether biomarker-based interventions or targeting the angiopoietin-2/renin pathway reduce clinical outcomes.

## Conclusions

In this biomarker study, angiopoietin-2 was significantly associated with all-cause mortality in OHCA patients who survived the first 48 h and may thus provide pathophysiological insight and improve risk stratification of these high-risk patients.

## Supplementary Information

Supplementary Information
