# Supplementary material for: Relationship between markers of inflammation and hemodynamic stress and death in patients with out-of-hospital cardiac arrest
Source: Sci Rep. 2021 May 11;11:9954. doi: 10.1038/s41598-021-88474-3 (PMC8113496; doi:10.1038/s41598-021-88474-3)

# Appendix

**Relationship between markers of inflammation and hemodynamic stress and death in patients with out-of-hospital cardiac arrest**

**Short Title:** Inflammatory and Hemodynamic Biomarkers in OHCA Patients

Thomas A Zelniker, MD, MSc;^1^* Ziya Kaya, MD;^2^* Eva Gamerdinger, MD;^2^ Sebastian Spaich, MD;^2^ Jan Stiepak, MD;^2^ Evangelos Giannitsis, MD;^2^ Hugo A Katus, MD;^2^ Michael R Preusch, MD^2^

1. Division of Cardiology, Medical University of Vienna, Austria
2. Department of Cardiology, Angiology, and Pneumology, University Hospital Heidelberg, Germany

* Contributed equally

[Supplemental Tables: 3](#_Toc67571524)

[Supplemental Table 1: Patient Characteristics stratified by death 3](#_Toc67571525)

[Supplemental Table 2: Median (interquartile range) biomarker levels at 48 hours of patients with out-of-hospital cardiac arrest (OHCA) compared with clinically healthy controls. 4](#_Toc67571526)

[Supplemental Table 3: Correlation matrix of the tested biomarkers. 6](#_Toc67571527)

[Supplemental Table 4: Unadjusted hazard ratios for biomarkers measured at 48 hours and all-cause death. 7](#_Toc67571528)

[Supplemental Table 5: Median (interquartile range) biomarker levels on admission of patients with out-of-hospital cardiac arrest (OHCA) compared with clinically healthy controls. 9](#_Toc67571529)

[Supplemental Table 6: Unadjusted hazard ratios for biomarkers measured on admission and all-cause death. 11](#_Toc67571530)

[Supplemental Figures 13](#_Toc67571531)

[Supplemental Figure 1: Study flow diagram 13](#_Toc67571532)

[Supplemental Figure 2: Median (interquartile range) biomarker levels at 48 hours of patients with out-of-hospital cardiac arrest (OHCA) compared with apparently healthy patients. 14](#_Toc67571533)

[Supplemental Figure 3: Adjusted hazard ratios for biomarkers per 1-unit increase in standardized biomarker levels and all-cause death. 15](#_Toc67571534)

[Supplemental Figure 4: Adjusted hazard ratios for biomarkers per 1-unit increase in standardized biomarker levels and all-cause death. 16](#_Toc67571535)

### Supplemental Tables:

#### Supplemental Table 1: Patient Characteristics stratified by death

Continuous variables are reported as median and interquartile range.

Legend: eGFR = estimated glomerular filtrations rate; ROSC = return of spontaneous circulation, SAPS II score = Simplified Acute Physiology Score II.

| **Demographic Characteristics** | **OHCA**  **Survivors**  **(N=68)** | **OHCA Non-Survivors**  **(N=52)** | **P** |
| --- | --- | --- | --- |
| **Age**, median [IQR] | 59.0 [49.8, 68.0] | 69.5 [60.3, 76.0] | <0.001 |
| **Female sex**, n (%) | 12 (17.6%) | 17 (32.7%) | 0.091 |
| **First monitored heart rhythm (shockable)**, n (%) | 55 (83.3%) | 15 (29.4%) | <0.001 |
| **Bystander-initiated CPR**, n (%) | 51 (75.0%) | 32 (61.5%) | 0.17 |
| **Time to ROSC (min)**, median [IQR] | 20.0 [14.0, 31.0] | 25.0 [17.0, 30.0] | 0.17 |
| **Cardiac etiology (yes)**, n (%) | 54 (87.1) | 24 (52.2) | <0.001 |
| **SAPS II score on admission**, median [IQR] | 62.00 [54.00, 66.00] | 70.00 [63.75, 74.00] | <0.001 |
| **Targeted temperature management at 33°C**, n (%) | 59 (86.8) | 38 (73.1) | 0.098 |
| **eGFR at 48 hours (ml/min/1.73m^2^)**, median [IQR] | 74.45 [45.30, 100.22] | 35.00 [23.55, 67.25] | <0.001 |
| **Cerebral Performance Category ≤2 at 30 days,** n (%) | 12 (21.1) | 51 (98.1) | <0.001 |

#### Supplemental Table 2: Median (interquartile range) biomarker levels at 48 hours of patients with out-of-hospital cardiac arrest (OHCA) compared with clinically healthy controls.

Legend: IFN = interferon; IL = interleukin; IP = interferon gamma-induce protein; MCP = monocyte chemoattractant protein, MIP = macrophage inflammatory protein, MMP = matrix metallopeptidase, OHCA = out-of-hospital cardiac arrest, TNF = tumor necrosis factor, TWEAK = tumor necrosis factor-like weak inducer of apoptosis; VEGF = vascular endothelial growth factor

| **Biomarker** | **Clinically Healthy**  **Controls** | **OHCA**  **Patients** | **P-value** | **OHCA**  **Survivors** | **OHCA**  **Deceased** | **P-value** | **P-**  **Omnibus** |
| --- | --- | --- | --- | --- | --- | --- | --- |
| Angiopoietin-1 | 51.492 (50.185, 51.911) | 1.467 (0.496, 5.254) | <0.001 | 1.467 (0.477, 4.737) | 1.451 (0.496, 6.198) | 0.70 | <0.001 |
| Angiopoietin-2 | 0.759 (0.448, 1.019) | 7.327 (4.111, 13.423) | <0.001 | 4.688 (3.474, 8.543) | 12.501 (6.176, 19.465) | <0.001 | <0.001 |
| Endothelin-1 | 0.022 (0.019, 0.026) | 0.025 (0.021, 0.028) | 0.025 | 0.024 (0.021, 0.027) | 0.025 (0.022, 0.029) | 0.13 | 0.026 |
| Eotaxin | 0.086 (0.072, 0.100) | 0.109 (0.092, 0.137) | <0.001 | 0.109 (0.089, 0.124) | 0.109 (0.103, 0.137) | 0.12 | <0.001 |
| Granzyme B | 0.015 (0.011, 0.025) | 0.015 (0.009, 0.027) | 0.72 | 0.013 (0.004, 0.018) | 0.025 (0.013, 0.035) | <0.001 | <0.001 |
| IFNγ | 0.035 (0.027, 0.042) | 0.056 (0.045, 0.066) | <0.001 | 0.054 (0.042, 0.064) | 0.060 (0.050, 0.071) | 0.035 | <0.001 |
| IL-1α | 0.009 (0.009, 0.010) | 0.011 (0.010, 0.012) | <0.001 | 0.010 (0.010, 0.012) | 0.011 (0.011, 0.012) | <0.001 | <0.001 |
| IL-1ꞵ | 0.009 (0.008, 0.009) | 0.013 (0.011, 0.015) | <0.001 | 0.012 (0.011, 0.014) | 0.013 (0.011, 0.016) | 0.032 | <0.001 |
| IL-4Rα | 0.212 (0.187, 0.238) | 0.534 (0.388, 0.821) | <0.001 | 0.451 (0.353, 0.648) | 0.702 (0.507, 0.978) | <0.001 | <0.001 |
| IL-6 | 0.000 (0.000, 0.000) | 0.078 (0.028, 0.173) | <0.001 | 0.064 (0.027, 0.128) | 0.118 (0.040, 0.196) | 0.029 | <0.001 |
| IL-8 | 0.009 (0.007, 0.011) | 0.016 (0.009, 0.030) | <0.001 | 0.013 (0.009, 0.026) | 0.023 (0.015, 0.038) | 0.001 | <0.001 |
| IL-9 | 0.512 (0.416, 0.621) | 0.653 (0.284, 1.151) | 0.49 | 0.668 (0.284, 1.151) | 0.580 (0.289, 1.223) | 0.63 | 0.74 |
| IL-10 | 3.741 (2.525, 4.460) | 8.634 (5.938, 13.070) | <0.001 | 7.976 (5.384, 10.772) | 9.650 (7.930, 15.138) | 0.002 | <0.001 |
| IL-12p70 | 0.112 (0.099, 0.118) | 0.152 (0.132, 0.170) | <0.001 | 0.145 (0.128, 0.170) | 0.161 (0.144, 0.175) | 0.095 | <0.001 |
| IL-17A | 0.000 (0.000, 0.000) | 0.001 (0.000, 0.002) | <0.001 | 0.001 (0.000, 0.002) | 0.001 (0.000, 0.003) | 0.019 | <0.001 |
| IL-21 | 0.012 (0.007, 0.016) | 0.031 (0.025, 0.038) | <0.001 | 0.028 (0.023, 0.035) | 0.033 (0.029, 0.040) | 0.006 | <0.001 |
| IL-23 | 0.386 (0.358, 0.407) | 0.453 (0.154, 0.665) | 0.26 | 0.411 (0.123, 0.574) | 0.627 (0.235, 0.716) | 0.002 | 0.005 |
| IP-10 | 1.460 (1.208, 1.641) | 15.649 (8.218, 28.786) | <0.001 | 15.346 (8.698, 27.925) | 17.175 (8.216, 29.381) | 0.57 | <0.001 |
| MCP-1 | 0.016 (0.000, 0.081) | 0.214 (0.087, 0.490) | <0.001 | 0.192 (0.070, 0.393) | 0.278 (0.110, 0.850) | 0.054 | <0.001 |
| MIP-1α | 0.322 (0.322, 0.323) | 0.356 (0.326, 0.398) | <0.001 | 0.355 (0.323, 0.397) | 0.356 (0.355, 0.399) | 0.006 | <0.001 |
| MIP-1ꞵ | 0.216 (0.210, 0.223) | 0.227 (0.220, 0.240) | <0.001 | 0.226 (0.216, 0.237) | 0.232 (0.226, 0.243) | 0.012 | <0.001 |
| MMP-8 | 0.915 (0.565, 2.768) | 2.610 (1.459, 5.853) | <0.001 | 2.227 (1.389, 5.066) | 3.714 (2.014, 7.055) | 0.001 | <0.001 |
| MUC-16 | 0.014 (0.000, 0.028) | 0.028 (0.009, 0.062) | 0.008 | 0.021 (0.007, 0.037) | 0.049 (0.017, 0.125) | <0.001 | <0.001 |
| PIGF | 1.005 (0.618, 1.168) | 1.095 (0.752, 1.300) | 0.11 | 1.089 (0.741, 1.300) | 1.109 (0.832, 1.300) | 0.51 | 0.22 |
| Procalcitonin | 0.030 (0.028, 0.034) | 1.269 (0.281, 3.114) | <0.001 | 0.496 (0.105, 2.132) | 2.222 (1.127, 5.546) | <0.001 | <0.001 |
| Renin | 0.603 (0.554, 0.624) | 1.044 (0.724, 1.962) | <0.001 | 0.992 (0.679, 1.651) | 1.187 (0.731, 5.592) | 0.052 | <0.001 |
| Resistin | 14.740 (14.598, 14.927) | 22.956 (16.939, 31.293) | <0.001 | 19.490 (15.839, 24.955) | 28.560 (20.707, 33.546) | <0.001 | <0.001 |
| TNFα | 0.009 (0.008, 0.010) | 0.010 (0.007, 0.013) | 0.33 | 0.009 (0.006, 0.011) | 0.012 (0.008, 0.016) | 0.004 | 0.012 |
| TWEAK | 8.179 (6.722, 9.319) | 2.367 (1.591, 3.380) | <0.001 | 2.277 (1.626, 3.265) | 2.424 (1.547, 3.509) | 0.95 | <0.001 |
| VEGF | 0.025 (0.004, 0.052) | 0.015 (0.004, 0.020) | 0.080 | 0.013 (0.000, 0.018) | 0.016 (0.009, 0.026) | 0.026 | 0.023 |

#### Supplemental Table 3: Correlation matrix of the tested biomarkers.

|  | Angiopoietin-1 | Angiopoietin-2 | Endothelin-1 | Eotaxin | Granzyme B | IFN gamma | IL-1 alpha | IL-1 beta | IL-4R alpha | IL-6 | IL-8 | IL-9 | IL-10 | IL-12p70 | IL-17A | IL-21 | IL-23 | IP-10 | MCP-1 | MIP-1 alpha | MIP-1 beta | MMP-8 | MUC-16 | PIGF | Procalcitonin | Renin | Resistin | TNF alpha | TWEAK | VEGF |
| --- | --- | --- | --- | --- | --- | --- | --- | --- | --- | --- | --- | --- | --- | --- | --- | --- | --- | --- | --- | --- | --- | --- | --- | --- | --- | --- | --- | --- | --- | --- |
| Angiopoietin-1 | 1 | -0.05 | -0.02 | 0.06 | 0.1 | 0 | 0.22 | 0.12 | -0.1 | -0.02 | 0 | -0.01 | 0.03 | 0.05 | -0.08 | -0.03 | -0.02 | -0.06 | 0.01 | 0.09 | 0.17 | 0.08 | -0.08 | 0.11 | -0.06 | -0.02 | 0.02 | -0.04 | 0.04 | 0.33 |
| Angiopoietin-2 |  | 1 | 0.02 | 0.03 | 0.42 | 0.19 | 0.16 | 0.16 | 0.58 | 0.3 | 0.49 | 0.01 | 0.36 | 0.11 | 0.3 | 0.31 | 0.34 | 0.11 | 0.14 | 0.08 | 0.37 | 0.48 | 0.4 | -0.02 | 0.48 | 0.33 | 0.29 | 0.53 | -0.02 | 0.05 |
| Endothelin-1 |  |  | 1 | 0.5 | 0.22 | -0.02 | 0.4 | 0.34 | 0.06 | 0.27 | 0.27 | 0.11 | 0.28 | 0.24 | 0.01 | 0.27 | 0.14 | 0.32 | 0.42 | 0.36 | 0.44 | 0.22 | 0.09 | 0.17 | 0.16 | 0.21 | 0.36 | 0.05 | -0.23 | 0.12 |
| Eotaxin |  |  |  | 1 | 0.22 | -0.01 | 0.46 | 0.48 | 0.15 | 0.31 | 0.39 | 0.12 | 0.31 | 0.3 | 0.13 | 0.33 | 0.12 | 0.19 | 0.34 | 0.48 | 0.5 | 0.27 | 0.07 | 0.15 | 0.19 | 0.45 | 0.43 | 0.04 | -0.16 | 0.37 |
| Granzyme B |  |  |  |  | 1 | 0.68 | 0.25 | 0.5 | 0.33 | 0.31 | 0.55 | 0.32 | 0.36 | 0.4 | 0.35 | 0.52 | 0.11 | 0.18 | 0.38 | 0.14 | 0.29 | 0.45 | 0.31 | 0.15 | 0.4 | 0.21 | 0.38 | 0.45 | -0.04 | 0.17 |
| IFN gamma |  |  |  |  |  | 1 | 0.06 | 0.4 | 0.23 | 0.17 | 0.31 | 0.46 | 0.13 | 0.39 | 0.3 | 0.41 | -0.16 | 0.13 | 0.22 | -0.21 | 0.16 | 0.16 | 0.12 | 0.11 | 0.26 | 0.09 | 0.14 | 0.3 | -0.03 | -0.09 |
| IL-1 alpha |  |  |  |  |  |  | 1 | 0.32 | 0.22 | 0.23 | 0.25 | 0.14 | 0.3 | 0.28 | 0.16 | 0.3 | 0.12 | 0.11 | 0.27 | 0.4 | 0.57 | 0.31 | 0.19 | 0.12 | 0.32 | 0.41 | 0.47 | 0.08 | -0.23 | 0.25 |
| IL-1 beta |  |  |  |  |  |  |  | 1 | 0.21 | 0.41 | 0.51 | 0.44 | 0.35 | 0.49 | 0.39 | 0.3 | -0.02 | 0.25 | 0.49 | 0.31 | 0.35 | 0.38 | 0.16 | 0.02 | 0.34 | 0.2 | 0.54 | 0.14 | -0.34 | 0.31 |
| IL-4R alpha |  |  |  |  |  |  |  |  | 1 | 0.44 | 0.55 | 0.03 | 0.4 | 0.31 | 0.24 | 0.37 | 0.35 | 0.21 | 0.25 | 0.18 | 0.35 | 0.42 | 0.33 | 0.06 | 0.54 | 0.38 | 0.52 | 0.42 | -0.21 | 0.13 |
| IL-6 |  |  |  |  |  |  |  |  |  | 1 | 0.55 | 0.08 | 0.44 | 0.23 | 0.25 | 0.12 | 0.38 | 0.39 | 0.7 | 0.22 | 0.25 | 0.45 | 0.15 | -0.06 | 0.25 | 0.26 | 0.43 | 0.33 | -0.27 | 0.18 |
| IL-8 |  |  |  |  |  |  |  |  |  |  | 1 | 0.26 | 0.58 | 0.38 | 0.4 | 0.39 | 0.24 | 0.43 | 0.58 | 0.23 | 0.41 | 0.55 | 0.33 | -0.01 | 0.51 | 0.36 | 0.56 | 0.51 | -0.21 | 0.13 |
| IL-9 |  |  |  |  |  |  |  |  |  |  |  | 1 | 0.13 | 0.5 | 0.17 | 0.16 | -0.63 | 0.02 | 0.18 | -0.3 | 0.19 | 0.19 | 0.05 | 0.03 | 0.23 | 0.06 | 0.27 | -0.07 | -0.13 | -0.11 |
| IL-10 |  |  |  |  |  |  |  |  |  |  |  |  | 1 | 0.23 | 0.3 | 0.17 | 0.29 | 0.5 | 0.39 | 0.26 | 0.33 | 0.37 | 0.3 | 0.03 | 0.27 | 0.34 | 0.41 | 0.44 | -0.05 | 0.11 |
| IL-12p70 |  |  |  |  |  |  |  |  |  |  |  |  |  | 1 | 0.18 | 0.41 | -0.19 | 0.16 | 0.29 | 0.29 | 0.34 | 0.16 | 0.11 | 0.1 | 0.38 | 0.15 | 0.6 | 0.03 | -0.28 | 0.21 |
| IL-17A |  |  |  |  |  |  |  |  |  |  |  |  |  |  | 1 | 0.24 | 0.13 | 0.18 | 0.25 | 0.12 | 0.18 | 0.26 | 0.16 | 0.07 | 0.43 | 0.21 | 0.29 | 0.31 | -0.32 | 0.07 |
| IL-21 |  |  |  |  |  |  |  |  |  |  |  |  |  |  |  | 1 | 0.04 | 0.13 | 0.11 | 0.21 | 0.6 | 0.37 | 0.17 | 0.22 | 0.5 | 0.37 | 0.4 | 0.26 | -0.11 | 0.08 |
| IL-23 |  |  |  |  |  |  |  |  |  |  |  |  |  |  |  |  | 1 | 0.15 | 0.19 | 0.54 | 0.11 | 0.27 | 0.28 | -0.02 | 0.17 | 0.13 | 0.14 | 0.39 | 0.02 | 0.15 |
| IP-10 |  |  |  |  |  |  |  |  |  |  |  |  |  |  |  |  |  | 1 | 0.5 | 0.17 | 0.15 | 0.04 | 0.19 | -0.08 | 0.11 | 0.08 | 0.29 | 0.31 | -0.16 | 0.01 |
| MCP-1 |  |  |  |  |  |  |  |  |  |  |  |  |  |  |  |  |  |  | 1 | 0.23 | 0.21 | 0.27 | 0.02 | -0.16 | 0.24 | 0.24 | 0.4 | 0.27 | -0.33 | 0.21 |
| MIP-1 alpha |  |  |  |  |  |  |  |  |  |  |  |  |  |  |  |  |  |  |  | 1 | 0.27 | 0.17 | 0.23 | -0.02 | 0.24 | 0.11 | 0.55 | -0.07 | -0.17 | 0.48 |
| MIP-1 beta |  |  |  |  |  |  |  |  |  |  |  |  |  |  |  |  |  |  |  |  | 1 | 0.39 | 0.2 | 0.07 | 0.43 | 0.47 | 0.42 | 0.21 | -0.12 | 0.08 |
| MMP-8 |  |  |  |  |  |  |  |  |  |  |  |  |  |  |  |  |  |  |  |  |  | 1 | 0.32 | 0.1 | 0.51 | 0.25 | 0.47 | 0.31 | -0.1 | 0.08 |
| MUC-16 |  |  |  |  |  |  |  |  |  |  |  |  |  |  |  |  |  |  |  |  |  |  | 1 | -0.07 | 0.27 | 0.1 | 0.31 | 0.33 | 0.04 | 0.11 |
| PIGF |  |  |  |  |  |  |  |  |  |  |  |  |  |  |  |  |  |  |  |  |  |  |  | 1 | 0.07 | 0.17 | 0.09 | 0.14 | -0.01 | 0.01 |
| Procalcitonin |  |  |  |  |  |  |  |  |  |  |  |  |  |  |  |  |  |  |  |  |  |  |  |  | 1 | 0.34 | 0.63 | 0.34 | -0.21 | 0.14 |
| Renin |  |  |  |  |  |  |  |  |  |  |  |  |  |  |  |  |  |  |  |  |  |  |  |  |  | 1 | 0.31 | 0.23 | -0.15 | 0.13 |
| Resistin |  |  |  |  |  |  |  |  |  |  |  |  |  |  |  |  |  |  |  |  |  |  |  |  |  |  | 1 | 0.12 | -0.33 | 0.38 |
| TNF alpha |  |  |  |  |  |  |  |  |  |  |  |  |  |  |  |  |  |  |  |  |  |  |  |  |  |  |  | 1 | 0.06 | -0.07 |
| TWEAK |  |  |  |  |  |  |  |  |  |  |  |  |  |  |  |  |  |  |  |  |  |  |  |  |  |  |  |  | 1 | -0.34 |

#### Supplemental Table 4: Unadjusted hazard ratios for biomarkers measured at 48 hours and all-cause death.

Unadjusted hazard ratio (HR) are reported per 1 unit increase in standardized biomarker concentrations with unadjusted (P_unadjusted_) and Bonferonni-Holm corrected (P_B-H_) P-values.

Legend: IFN = interferon; IL = interleukin; IP = interferon gamma-induce protein; MCP = monocyte chemoattractant protein, MIP = macrophage inflammatory protein, MMP = matrix metallopeptidase, TNF = tumor necrosis factor, TWEAK = tumor necrosis factor-like weak inducer of apoptosis; VEGF = vascular endothelial growth factor

| **Biomarker** | **HR (95%-CI)** | **P_Unadjusted_** | **P_B-H_** |
| --- | --- | --- | --- |
| Angiopoietin-2 | 1.79 (1.46-2.19) | <0.001 | <0.001 |
| Procalcitonin | 1.73 (1.38-2.18) | <0.001 | <0.001 |
| Resistin | 2.09 (1.54-2.84) | <0.001 | <0.001 |
| IL-4Rα | 1.52 (1.27-1.83) | <0.001 | <0.001 |
| MMP-8 | 1.36 (1.15-1.61) | <0.001 | 0.010 |
| TNFα | 1.29 (1.11-1.49) | <0.001 | 0.020 |
| Renin | 1.36 (1.12-1.64) | 0.002 | 0.045 |
| IL-1α | 1.29 (1.10-1.52) | 0.002 | 0.046 |
| MCP-1 | 1.36 (1.11-1.66) | 0.003 | 0.060 |
| IL-23 | 1.47 (1.14-1.89) | 0.003 | 0.060 |
| MIP-1β | 1.35 (1.11-1.65) | 0.003 | 0.062 |
| IL-21 | 1.59 (1.16-2.18) | 0.004 | 0.085 |
| IL-8 | 1.25 (1.06-1.48) | 0.009 | 0.17 |
| IL-6 | 1.26 (1.06-1.50) | 0.010 | 0.17 |
| IFNγ | 1.40 (1.07-1.81) | 0.012 | 0.20 |
| IL-17A | 2.69 (1.24-5.86) | 0.013 | 0.20 |
| VEGF | 1.44 (1.05-1.98) | 0.024 | 0.33 |
| IP-10 | 1.25 (1.02-1.51) | 0.028 | 0.36 |
| Granzyme B | 1.16 (0.99-1.36) | 0.061 | 0.73 |
| MIP-1α | 1.29 (0.97-1.73) | 0.079 | 0.87 |
| MUC-16 | 1.15 (0.97-1.36) | 0.11 | 1.00 |
| Endothelin-1 | 1.18 (0.95-1.47) | 0.13 | 1.00 |
| IL-12p70 | 1.18 (0.90-1.54) | 0.22 | 1.00 |
| IL-1β | 1.07 (0.91-1.26) | 0.39 | 1.00 |
| IL-9 | 1.18 (0.78-1.77) | 0.43 | 1.00 |
| TWEAK | 0.85 (0.56-1.29) | 0.44 | 1.00 |
| Angiopoietin-1 | 1.30 (0.66-2.57) | 0.44 | 1.00 |
| PIGF | 1.08 (0.78-1.49) | 0.64 | 1.00 |
| IL-10 | 1.02 (0.84-1.24) | 0.80 | 1.00 |
| Eotaxin | 1.01 (0.79-1.29) | 0.92 | 1.00 |

#### Supplemental Table 5: Median (interquartile range) biomarker levels on admission of patients with out-of-hospital cardiac arrest (OHCA) compared with clinically healthy controls.

Legend: IFN = interferon; IL = interleukin; IP = interferon gamma-induce protein; MCP = monocyte chemoattractant protein, MIP = macrophage inflammatory protein, MMP = matrix metallopeptidase, OHCA = out-of-hospital cardiac arrest, TNF = tumor necrosis factor, TWEAK = tumor necrosis factor-like weak inducer of apoptosis; VEGF = vascular endothelial growth factor

| **Biomarker** | **Clinically Healthy**  **Controls** | **OHCA**  **Patients** | **P-value** | **OHCA**  **Survivors** | **OHCA**  **Deceased** | **P-value** | **P-**  **Omnibus** |
| --- | --- | --- | --- | --- | --- | --- | --- |
| Angiopoietin-1 | 51.492 (50.185, 51.911) | 10.273 (5.238, 17.942) | <0.001 | 10.798 (6.658, 20.694) | 9.565 (4.672, 16.902) | 0.36 | <0.001 |
| Angiopoietin-2 | 0.759 (0.448, 1.019) | 3.906 (2.342, 6.754) | <0.001 | 3.189 (1.748, 4.860) | 6.261 (3.388, 10.459) | <0.001 | <0.001 |
| Endothelin-1 | 0.022 (0.019, 0.026) | 0.023 (0.021, 0.025) | 0.11 | 0.023 (0.020, 0.026) | 0.023 (0.021, 0.025) | 0.66 | 0.26 |
| Eotaxin | 0.086 (0.072, 0.100) | 0.120 (0.098, 0.153) | <0.001 | 0.119 (0.098, 0.153) | 0.120 (0.103, 0.153) | 0.99 | <0.001 |
| Granzyme B | 0.015 (0.011, 0.025) | 0.032 (0.016, 0.051) | <0.001 | 0.028 (0.016, 0.046) | 0.035 (0.018, 0.053) | 0.32 | <0.001 |
| IFN | 0.035 (0.027, 0.042) | 0.053 (0.045, 0.063) | <0.001 | 0.052 (0.043, 0.059) | 0.055 (0.046, 0.067) | 0.23 | <0.001 |
| IL-1a | 0.009 (0.009, 0.010) | 0.010 (0.010, 0.011) | <0.001 | 0.010 (0.009, 0.011) | 0.011 (0.010, 0.011) | 0.21 | <0.001 |
| IL-1 | 0.009 (0.008, 0.009) | 0.012 (0.011, 0.014) | <0.001 | 0.013 (0.011, 0.014) | 0.012 (0.011, 0.014) | 0.87 | <0.001 |
| IL-4Ra | 0.212 (0.187, 0.238) | 0.369 (0.289, 0.493) | <0.001 | 0.309 (0.253, 0.413) | 0.424 (0.359, 0.602) | <0.001 | <0.001 |
| IL-6 | 0.000 (0.000, 0.000) | 0.055 (0.020, 0.171) | <0.001 | 0.036 (0.010, 0.106) | 0.098 (0.035, 0.242) | <0.001 | <0.001 |
| IL-8 | 0.009 (0.007, 0.011) | 0.034 (0.017, 0.069) | <0.001 | 0.026 (0.015, 0.051) | 0.045 (0.021, 0.080) | 0.019 | <0.001 |
| IL-9 | 0.512 (0.416, 0.621) | 0.729 (0.219, 1.151) | 0.091 | 0.749 (0.351, 1.169) | 0.726 (0.201, 1.106) | 0.52 | 0.18 |
| IL-10 | 3.741 (2.525, 4.460) | 20.060 (8.860, 41.304) | <0.001 | 15.124 (7.412, 31.700) | 26.708 (15.047, 63.712) | 0.002 | <0.001 |
| IL-12p70 | 0.112 (0.099, 0.118) | 0.146 (0.129, 0.163) | <0.001 | 0.145 (0.128, 0.163) | 0.150 (0.129, 0.163) | 0.65 | <0.001 |
| IL-17A | 0.000 (0.000, 0.000) | 0.000 (0.000, 0.001) | <0.001 | 0.000 (0.000, 0.001) | 0.000 (0.000, 0.001) | 0.15 | <0.001 |
| IL-21 | 0.012 (0.007, 0.016) | 0.025 (0.021, 0.030) | <0.001 | 0.025 (0.020, 0.029) | 0.026 (0.023, 0.031) | 0.090 | <0.001 |
| IL-23 | 0.386 (0.358, 0.407) | 0.444 (0.172, 0.667) | 0.096 | 0.386 (0.140, 0.585) | 0.585 (0.262, 0.716) | 0.006 | 0.005 |
| IP-10 | 1.460 (1.208, 1.641) | 7.549 (3.308, 14.311) | <0.001 | 7.158 (3.435, 15.126) | 7.844 (3.308, 13.710) | 0.98 | <0.001 |
| MCP-1 | 0.016 (0.000, 0.081) | 0.270 (0.106, 0.714) | <0.001 | 0.318 (0.110, 0.877) | 0.241 (0.096, 0.578) | 0.21 | <0.001 |
| MIP-1a | 0.322 (0.322, 0.323) | 0.355 (0.325, 0.398) | <0.001 | 0.354 (0.323, 0.394) | 0.357 (0.354, 0.399) | 0.005 | <0.001 |
| MIP-1 | 0.216 (0.210, 0.223) | 0.224 (0.219, 0.233) | <0.001 | 0.222 (0.216, 0.233) | 0.226 (0.219, 0.240) | 0.25 | <0.001 |
| MMP-8 | 0.915 (0.565, 2.768) | 3.599 (2.082, 9.587) | <0.001 | 2.839 (1.821, 9.256) | 4.184 (2.106, 9.587) | 0.47 | <0.001 |
| MUC-16 | 0.014 (0.000, 0.028) | 0.024 (0.008, 0.056) | <0.001 | 0.020 (0.007, 0.031) | 0.034 (0.010, 0.105) | 0.008 | <0.001 |
| PIGF | 1.005 (0.618, 1.168) | 0.872 (0.685, 1.013) | 0.020 | 0.867 (0.685, 0.995) | 0.881 (0.694, 1.021) | 0.91 | 0.065 |
| Procalcitonin | 0.030 (0.028, 0.034) | 0.077 (0.038, 0.173) | <0.001 | 0.059 (0.030, 0.131) | 0.101 (0.048, 0.262) | 0.020 | <0.001 |
| Renin | 0.603 (0.554, 0.624) | 0.609 (0.514, 1.105) | 0.65 | 0.633 (0.511, 1.037) | 0.600 (0.523, 1.256) | 0.52 | 0.86 |
| Resistin | 14.740 (14.598, 14.927) | 16.640 (14.946, 21.642) | <0.001 | 16.028 (14.071, 20.211) | 17.331 (16.374, 24.350) | 0.002 | <0.001 |
| TNFa | 0.009 (0.008, 0.010) | 0.008 (0.006, 0.010) | 0.002 | 0.007 (0.005, 0.010) | 0.008 (0.006, 0.011) | 0.28 | 0.005 |
| TWEAK | 8.179 (6.722, 9.319) | 5.296 (3.506, 38.457) | 0.019 | 6.930 (3.543, 40.726) | 4.618 (3.372, 20.131) | 0.19 | 0.011 |
| VEGF | 0.025 (0.004, 0.052) | 0.007 (0.000, 0.018) | <0.001 | 0.004 (0.000, 0.015) | 0.009 (0.000, 0.019) | 0.13 | <0.001 |

#### Supplemental Table 6: Unadjusted hazard ratios for biomarkers measured on admission and all-cause death.

Unadjusted hazard ratio (HR) are reported per 1 unit increase in standardized biomarker concentrations with unadjusted (P_unadjusted_) and Bonferonni-Holm corrected (P_B-H_) P-values.

Legend: IFN = interferon; IL = interleukin; IP = interferon gamma-induce protein; MCP = monocyte chemoattractant protein, MIP = macrophage inflammatory protein, MMP = matrix metallopeptidase, TNF = tumor necrosis factor, TWEAK = tumor necrosis factor-like weak inducer of apoptosis; VEGF = vascular endothelial growth factor

| **Biomarker** | **HR (95%-CI)** | **P_Unadjusted_** | **P_B-H_** |
| --- | --- | --- | --- |
| Angiopoietin-2 | 1.29 (1.11-1.49) | <0.001 | 0.019 |
| Resistin | 1.38 (1.10-1.74) | 0.006 | 0.17 |
| IL-23 | 1.24 (1.03-1.50) | 0.022 | 0.61 |
| IL-4Rα | 1.22 (1.01-1.48) | 0.036 | 0.96 |
| MUC-16 | 1.19 (0.99-1.42) | 0.063 | 1.00 |
| IL-6 | 1.19 (0.99-1.43) | 0.071 | 1.00 |
| MIP-1α | 1.29 (0.97-1.72) | 0.077 | 1.00 |
| IL-21 | 1.30 (0.96-1.75) | 0.084 | 1.00 |
| IL-10 | 1.20 (0.97-1.47) | 0.085 | 1.00 |
| Granzyme B | 1.17 (0.98-1.41) | 0.088 | 1.00 |
| Renin | 1.15 (0.97-1.36) | 0.11 | 1.00 |
| MCP-1 | 0.81 (0.57-1.14) | 0.23 | 1.00 |
| TNFα | 1.12 (0.93-1.35) | 0.24 | 1.00 |
| IL-1α | 1.15 (0.90-1.46) | 0.26 | 1.00 |
| VEGF | 1.17 (0.87-1.57) | 0.29 | 1.00 |
| Eotaxin | 0.78 (0.49-1.25) | 0.31 | 1.00 |
| TWEAK | 0.87 (0.65-1.15) | 0.32 | 1.00 |
| IFNγ | 1.16 (0.86-1.55) | 0.33 | 1.00 |
| Procalcitonin | 1.10 (0.90-1.33) | 0.35 | 1.00 |
| MMP-8 | 1.11 (0.89-1.40) | 0.36 | 1.00 |
| Angiopoietin-1 | 0.80 (0.48-1.33) | 0.39 | 1.00 |
| IL-8 | 1.06 (0.88-1.29) | 0.54 | 1.00 |
| PIGF | 0.92 (0.65-1.29) | 0.62 | 1.00 |
| IL-17A | 1.26 (0.50-3.18) | 0.63 | 1.00 |
| IL-9 | 0.91 (0.61-1.37) | 0.66 | 1.00 |
| MIP-1β | 1.03 (0.82-1.30) | 0.77 | 1.00 |
| IL-12p70 | 1.04 (0.79-1.37) | 0.77 | 1.00 |
| IP-10 | 0.97 (0.74-1.26) | 0.80 | 1.00 |
| IL-1β | 0.97 (0.76-1.24) | 0.81 | 1.00 |
| Endothelin-1 | 1.00 (0.78-1.27) | 0.99 | 1.00 |

### Supplemental Figures

#### Supplemental Figure 1: Study flow diagram


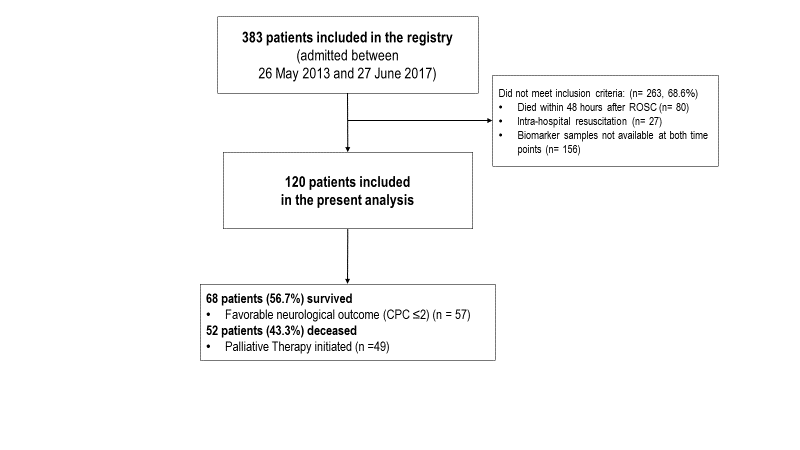


#### Supplemental Figure 2: Median (interquartile range) biomarker levels at 48 hours of patients with out-of-hospital cardiac arrest (OHCA) compared with apparently healthy patients.

Bonferroni-Holm corrected p-values for pairwise Wilcoxon Rank sum tests reported unless Kruskal Wallis rank sum test >0.05.

Legend: IFN = interferon; IL = interleukin; IP = interferon gamma-induce protein; MCP = monocyte chemoattractant protein, MIP = macrophage inflammatory protein, MMP = matrix metallopeptidase, TNF = tumor necrosis factor, TWEAK = tumor necrosis factor-like weak inducer of apoptosis; VEGF = vascular endothelial growth factor


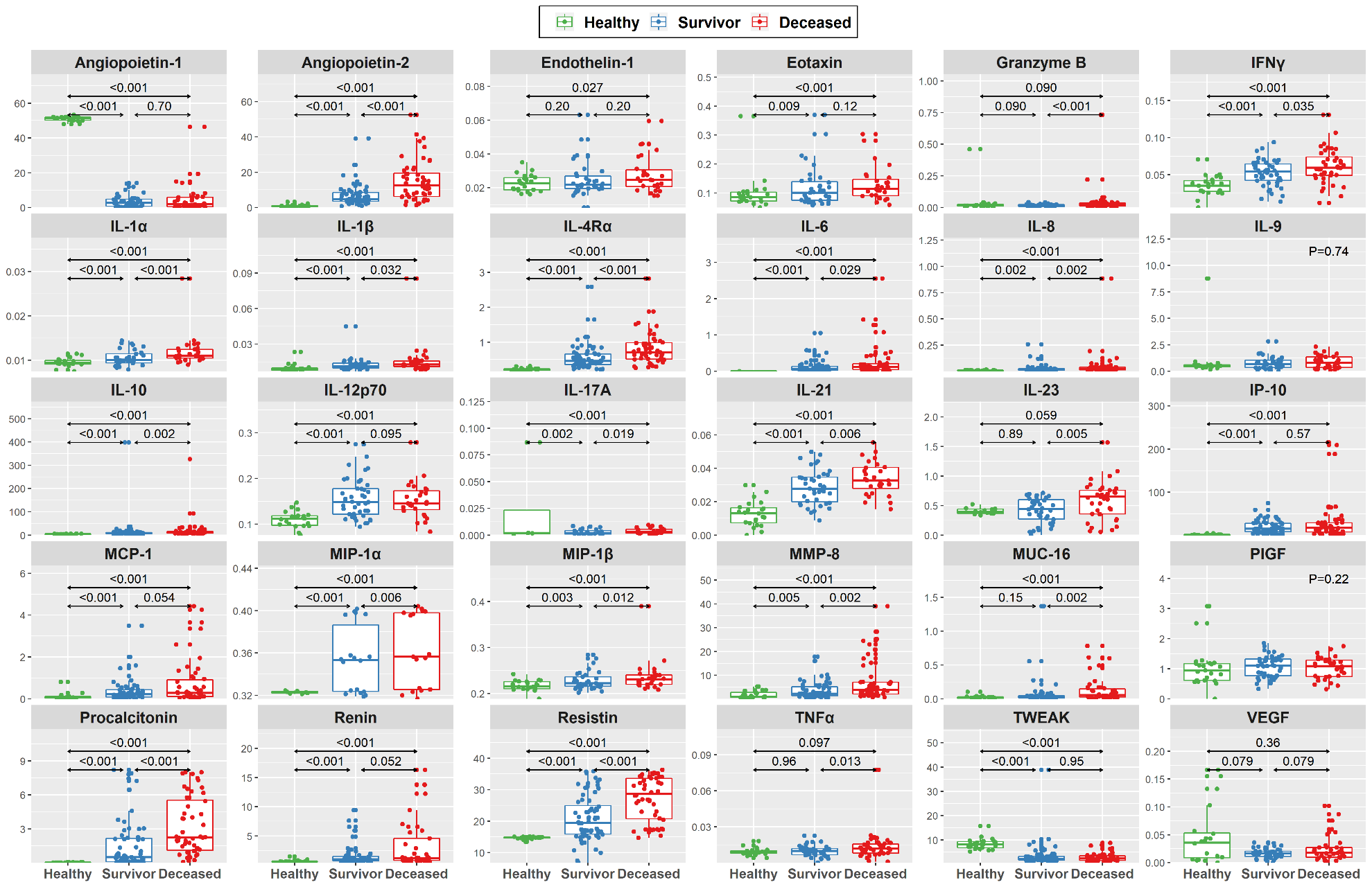


#### Supplemental Figure 3: Adjusted hazard ratios for biomarkers per 1-unit increase in standardized biomarker levels and all-cause death.

The models were adjusted for age, sex, estimated glomerular filtration rate at 48 hours, lactate levels at 48 hours, baseline Simplified Acute Physiology Score II score, bystander resuscitation, presence of shockable rhythm as the first monitored heart rhythm, and type of targeted temperature management (33**°** versus 36°Celsius).

**
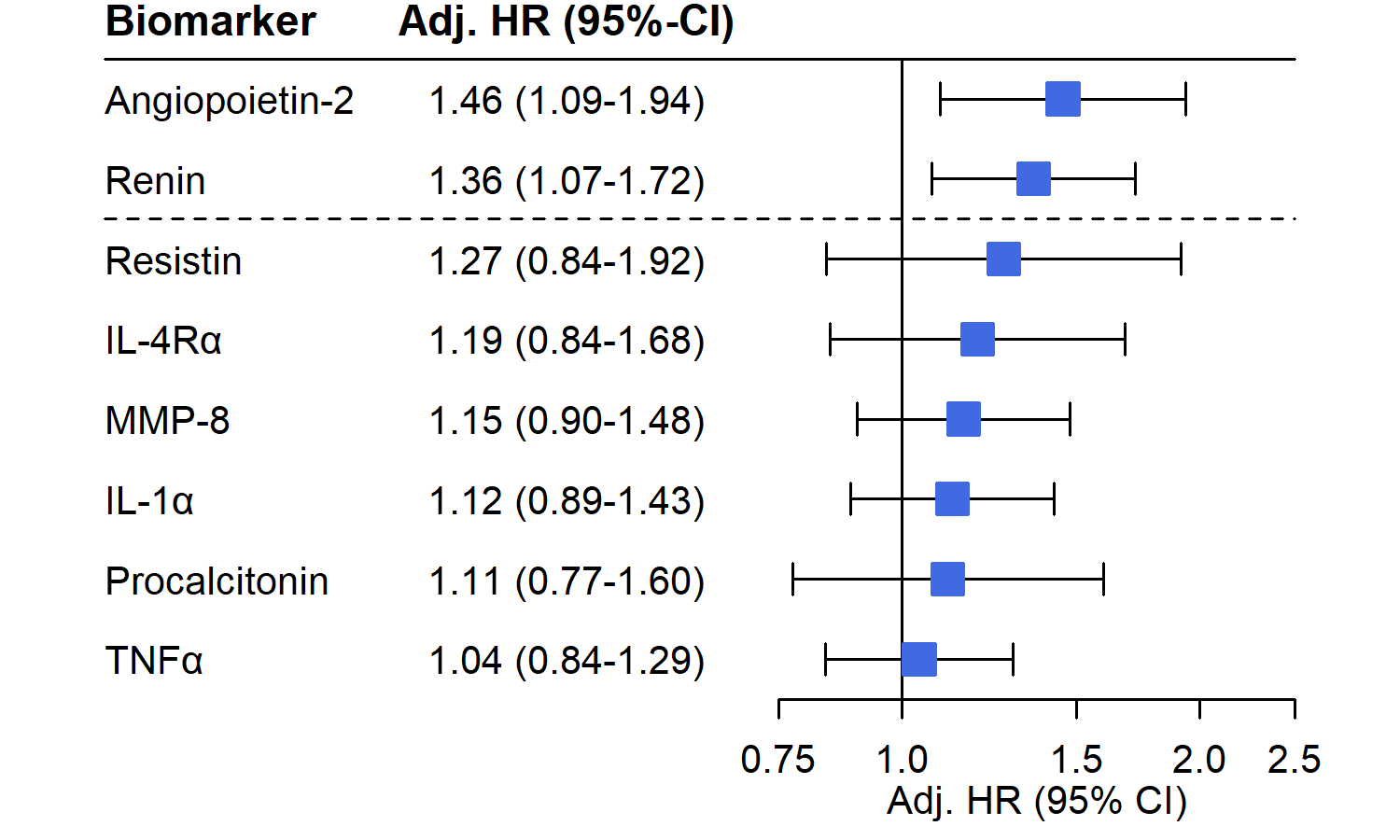
**

#### Supplemental Figure 4: Adjusted hazard ratios for biomarkers per 1-unit increase in standardized biomarker levels and all-cause death.

The models were adjusted for age, sex, estimated glomerular filtration rate at 48 hours, lactate levels at 48 hours, baseline Simplified Acute Physiology Score II score, baseline high-sensitivity troponin T, baseline high-sensitivity CRP, bystander resuscitation, presence of shockable rhythm as the first monitored heart rhythm, and type of targeted temperature management (33**°** versus 36°Celsius).


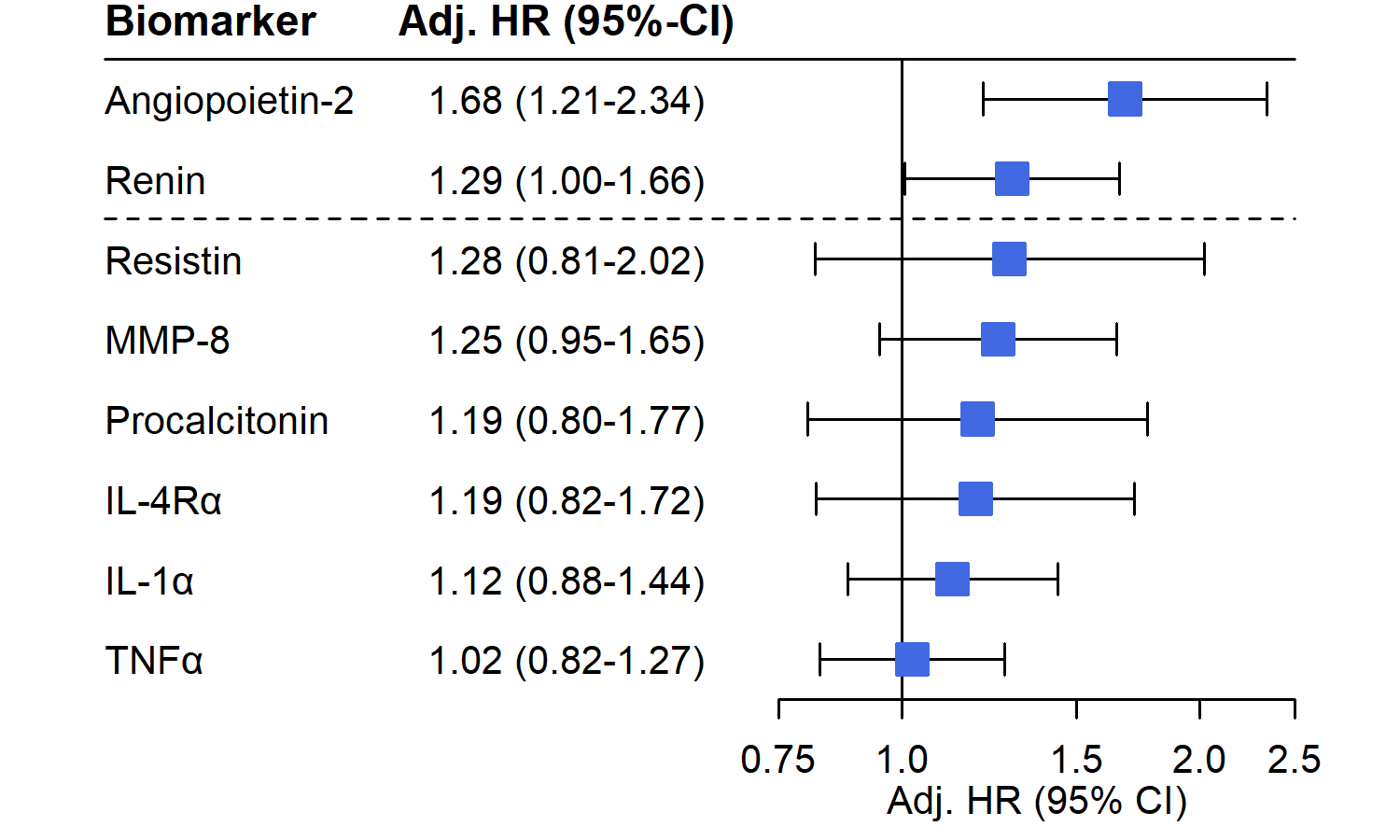

Supplement: Supplementary file 1 — Supplementary Information [file 41598_2021_88474_MOESM1_ESM.docx]
